# Retraction: Molecular structure and target recognition of neuronal calcium sensor proteins

**DOI:** 10.3389/fnmol.2016.00038

**Published:** 2016-05-20

**Authors:** Frontiers in Neuroscience Editorial Office

The authors and the journal retract the 9 February 2012 article cited above. This article is a duplicate of an article that had been previously submitted for publication in another journal [Molecular structure and target recognition of neuronal calcium sensor proteins, Ames, J. B., Lim, S., *Biochim. Biophys. Acta*. 2012 Aug; 1820 (8):1205–1213. doi: 10.1016/j.bbagen.2011.10.003. Epub 2011 Oct 13. Review. PMID: 22020049]. The authors regret not having detected their error and apologise to the readers of the Journal for any confusion this may have caused.

